# Introducing virtual reality therapy for inpatients with dementia admitted to an acute care hospital: learnings from a pilot to pave the way to a randomized controlled trial

**DOI:** 10.1186/s40814-020-00708-9

**Published:** 2020-10-31

**Authors:** Lora Appel, Erika Kisonas, Eva Appel, Jennifer Klein, Deanna Bartlett, Jarred Rosenberg, Christopher Smith

**Affiliations:** 1 Faculty of Health, School of Health Policy and Management, York University, Toronto, Ontario Canada; 2 OpenLab, University Health Network, Toronto, Ontario Canada; 3 Michael Garron Hospital, Toronto, Ontario Canada

**Keywords:** Non-pharmacological therapy, Dementia, Head-mounted display, Interventional study, Protocol, Simulation, Acute care, BPSD, Virtual reality

## Abstract

**Background:**

Behavioural and psychological symptoms of dementia (BPSD) are difficult to manage, particularly in acute care settings. As virtual reality (VR) technology becomes increasingly accessible and affordable, there is growing interest among clinicians to evaluate VR therapy in hospitalized patients, as an alternative to administering antipsychotics/sedatives or using physical restraints associated with negative side effects.

**Objectives:**

Validate and refine the proposed research protocol for a randomized controlled trial (RCT) that evaluates the impact of VR therapy on managing BPSD in acute care hospitals. Special attention was given to ascertain the processes of introducing non-pharmacological interventions in acute care hospitals.

**Methods:**

Ten patients 65 years or older (mean = 87) previously diagnosed with dementia, admitted to an acute care hospital, were recruited over 3-month period into a prospective longitudinal pilot study. The intervention consisted of viewing 20-min of immersive 360° VR using a head-mounted display. Baseline and outcomes data were collected from the hospital electronic medical records, pre/post mood-state questionnaires, Neuropsychiatric Inventory (NPI) score, and standardized qualitative observations. Comprehensive process data and workflow were documented, including timestamps for each study task and detailed notes on personnel requirements and challenges encountered.

**Results:**

Of 516 patients admitted during the study, 67 met the inclusion/exclusion criteria. In total, 234 calls were initiated to substitute decision makers (SDM) of the 67 patients for the consenting process. Nearly half (45.6%) of SDMs declined participation, and 40% could not be reached in time before patients being discharged, resulting in 57 eligible patients not being enrolled. Ten consented participants were enrolled and completed the study. The initial VR session averaged 53.6 min, largely due to the administration of NPI (mean = 19.5 min). Only four participants were able to respond reliably to questions. Seven participants opted for additional VR therapy sessions; of those providing feedback regarding the VR content, they wanted more varied scenery (animals, fields of flowers, holiday themes). Few sessions (4/18) encountered technical difficulties.

**Conclusion:**

The pilot was instrumental in identifying issues and providing recommendations for the RCT. Screening, inclusion criteria, consenting, data collection, and interaction with SDMs and hospital staff were all processes requiring changes and optimizations. Overall, patients with dementia appear to tolerate immersive VR, and with suggested protocol alterations, it is feasible to evaluate this non-pharmacological intervention in acute care hospitals.

**Supplementary Information:**

The online version contains supplementary material available at 10.1186/s40814-020-00708-9.

## Key messages on feasibility

1. What uncertainties about feasibility existed prior to this study?

There is growing interest in using VR-based therapeutic interventions for people with dementia in different settings, including long-term care and rehab facilities, community care/private homes, and acute care hospitals. There was uncertainty about whether and how VR therapy interventions can be introduced and evaluated in acute care hospitals, where it was strongly desired for its potential to help deprescribe antipsychotics for the management of responsive behaviours and other symptoms of dementia.

The following factors, among others, were challenging for the study feasibility in the acute care hospital:

- Nature of this fast-paced and busy environment, packed with daily medical tests and procedures with variable last-minute changing schedules
- Strict hygiene requirements for the VR equipment
- Hospital staff focused mainly on pharmacologic solutions (e.g. administering antipsychotics/ sedatives),
- Acutely ill patient participants with all stages of dementia, presenting with complex comorbidities and increased frailty,
- Substitute decision makers (SDMs) not easily reachable to provide consent for patient participation during their hospitalization (usually a relatively short length of stay).
- Validated instruments for studying changes in patients with dementia were not designed for short stays such as in acute care hospitals

(2) What are the key feasibility findings from this study?

The pilot was instrumental in identifying issues and providing recommendations for conducting the subsequent randomized controlled trial (RCT). Screening patients, inclusion criteria, consenting/ assenting, data collection tools, and interaction with SDMs and hospital staff were among processes, materials, and protocols that required changes and optimizations.

(3) What are the implications of the feasibility findings on the design of the main study?

A VR therapy intervention can be evaluated in acute care hospitals if suggested protocol alterations are implemented. Overall, patients with dementia appear to accept immersive VR, and there is a need to conduct rigorous studies and establish guidelines to ensure reliability and consistency in evaluating VR interventions. Our research team has since implemented the protocol changes resulting from the pilot study and we have successfully started recruitment for an RCT at a teaching hospital in downtown Toronto.

## Background

Behavioural and psychological symptoms of dementia (BPSD) are common in individuals with dementia and are particularly difficult to manage in acute care settings. Resident responsive behaviours have been shown to relate to staff burnout levels [1] which contribute to high absenteeism and turnover rate, low engagement, and higher risk of abuse or neglect incidents [2, 3]. Such workplace violence is a widespread problem that many health systems have struggled to manage [4], and need for supports in the community is ever increasing [5].

To date, most interventions used to manage BPSD include medications (neuroleptic/sedating medications) and application of physical barriers and restraints (alarms, locks, Buxton chairs, tethers), both of which raise ethical concerns and have been associated with hastening of cognitive and physical decline. Several non-pharmacological approaches have also been tried with varying levels of success [6–9]. For example, multidisciplinary care, massage and touch therapy, and music combined with massage and touch therapy were clinically more efficacious than usual care in reducing combined agitation and aggression, and outdoor activities were more efficacious than antipsychotics for treating physical aggression [6]. An earlier review of the literature [7] which studied the effectiveness among seven types of nonpharmacological interventions for agitation in older adults with dementia (sensory intervention, social contact, activities, environmental modification, caregiver training, combination therapy, and behavioural therapy) found only “sensory interventions” (aromatherapy, thermal bath, and calming music and hand massage) to be statistically significantly effective in reducing agitation. However, these have not been widely adopted; usually due to difficulties in implementation across the spectrum of care [10]. Articles exploring the research process of non-pharmacological interventions, such as hand massage [11], music therapy [12], or pet therapy [13, 14] for patients with dementia discuss factors that affect the study protocol including intervention and environmental factors. Their authors provide suggestions such as having multiple researchers and adding a qualitative component to record the participant’s reactions [10].

Virtual reality (VR) is a computer-generated or computer-simulated three-dimensional environment that synchronously stimulates our senses (vision, hearing, touch, smell) to create the illusion of reality that closely resembles the physical world. Level of immersion has proven important in the application of VR for the treatment of phobias (acrophobia, aviophobia, arachnophobia), anxiety (social anxiety disorder, public speaking anxiety), panic disorder, posttraumatic stress disorder, and substance abuse disorders (alcohol and nicotine), drawing principles from cognitive-behavioural and exposure therapy techniques [15]. VR has also been used for physical and neuro-rehabilitation and pain reduction treatments [16]. More recently, evidence suggests that VR therapy may alleviate stress, depression, anxiety, and feelings of isolation in institutionalized older adults [17–20]. However, most of these accounts are not grounded in systematic research and therefore do not result in high-quality evidence necessary for broader support, investment, and implementation of VR as an alternative non-pharmacological intervention for managing BPSD.

A growing number of studies show a connection between exposure to natural environments (seeing greenery, hearing outside natural sounds) and better mental health. Exposure to nature, even when virtually, results in benefits such as reduced depression, stress, and anxiety. Virtual environments offer a methodology for presenting digitally recreated simulations of the real world with the potential of enhancing ecological validity while maintaining experimental control in social neuroscience research [21].

Given the growing interest in therapeutic VR, there is a consensus among leaders in the field that standardized evaluation methodology and implementation guidelines are sorely needed. A recent article, published by an international working group in the field (Virtual Reality Committee of Outcomes Research Experts (VR-CORE)), recommends that VR trials follow a 3-phase framework based on the Food and Drug Administration Phase I-III pharmacotherapy model [22]. VR1 studies focus on content development by working with patient and provider end-users through principles of human-centred design; VR2 trials conduct early testing with a focus on feasibility, acceptability, tolerability, and initial clinical efficacy; and VR3 trials are RCTs that evaluate clinically important outcomes versus a control condition [22]. While the VR-CORE group brings the necessary theoretical framework to conceptualize VR studies, gaps remain in the provisioning of detailed guidelines to aid with designing and conducting these studies. Our study was aimed at documenting the process, identifying challenges, and providing recommendations for conducting therapeutic VR studies for patients with dementia in acute care settings.

The reflections of the investigators may assist other researchers to overcome obstacles in introducing and evaluating VR and other non-pharmacological interventions for people with dementia, both within acute and long-term care settings.

## Objectives

The primary objective of the pilot study was to inform the design of a subsequent RCT and evaluate the feasibility of the proposed protocol. Special attention was given to validating enrollment and data collection processes (e.g. obtaining informed consent, conducting interviews with participants), validating proposed instruments (questionnaires, interviews, qualitative observation script), documenting issues with equipment, and identifying timing and personnel requirements, including potential changes to clinical workflow.

Secondary objectives were to explore the tolerability, comfort and safety, and the impact on wellbeing (enjoyment, relaxation, engagement, reminiscence) of the VR intervention on patients with dementia admitted to an acute care hospital. The findings related to secondary objectives are described in detail in another manuscript [23].

### Research question

Given the current standard practices in acute care hospitals (including workflow, processes, materials, resources), is it feasible to administer VR therapy to inpatients with dementia, as a non-pharmacological therapeutic approach to manage BPSD?

## Methods

### Design

This prospective, longitudinal study was conducted at Michael Garron Hospital (MGH), a community teaching hospital located in Toronto, Canada, in collaboration with OpenLab, an innovation centre at University Health Network. Data were collected between July 31, 2018, and October 31, 2018, using a mixed-methods (quantitative and qualitative) research approach. From the electronic medical records (EMR), the team collected physiological markers (e.g. blood pressure, heart rate, respiratory rate, blood glucose), delirium status, factors related to the hospital care experience (instances of wandering, insomnia, pressure ulcers, falls), hospital length of stay, discharge disposition, in-hospital mortality, demographics, and diagnoses (cognitive assessments, comorbidities, general health history). In addition, the research team conducted pre- and post-VR mood state questionnaires, NPI, and recorded qualitative observations during the study sessions. A modified version of the State-Trait Anxiety Inventory (STAI Y) [24] was used to collect information about participant’s current state of anxiety pre- and post-intervention. Post VR therapy, open-ended questions were asked to capture feedback about any discomfort experienced: whether the head-mounted display (HMD) was too heavy, if it applied too much pressure on their head, face, or nose, and sound quality and image focus. A modified version of the Music in Dementia Assessment Scales (MiDAS), developed and validated to evaluate music therapy for people with dementia [25], was completed by the research coordinator (RC) to assess whether there were observable changes in the participant’s mood/behaviour and engagement (e.g. interest, response, enjoyment) while exposed to VR therapy. The RC recorded any vocalizations, changes in facial expressions, breathing patterns, gestures, body movements, and level of activity. Caregiver feedback regarding participant response to the VR intervention was also recorded. This included caregiver insights as to why participants reacted in certain ways to certain VR films.

Ethics approval was received from MGH Research Ethics Board (REB ref 748-1806-Mis-321 dated June 26, 2018); informed consent for study participation was obtained for all participants through their SDMs, and assent was obtained prior to each study session from participants themselves.

### Participants

Participants were screened and recruited sequentially, daily within a 3-month period (excluding weekends and statutory holidays). *Inclusion* criteria required that participants were aged 65 years or older, with documented diagnosis of dementia, and admitted as an inpatient at MGH. Patients were *excluded* if they had open facial wounds, cervical conditions that would make use of a VR headset unsafe, or no contactable substitute decision maker. Table 1 describes baseline demographic and clinical characteristics of the ten recruited participants. Ten participants were considered sufficient for this pilot as we were validating study protocol and were not seeking statistical significance of clinical outcomes.

**Table 1:** Demographic and baseline information

	Participants (*n* = 10)
	Frequency
Age (years)^a^	86.5 (5.68)
Gender	
Male	2
Female	8
Dementia type diagnosis	
Mixed dementia	4
Alzheimer’s dementia	4
Vascular dementia	1
Frontotemporal dementia	1
Dementia stages	
Mild	2
Moderate	1
Advanced	4
Unspecified	3
Delirium diagnosis	
No delirium	4
Sub-acute	1
Acute	3
Chronic	0
Unspecified	2
Primary language	
English	5
Greek/Macedonian	3
Bengali	1
Chinese	1
Current living state	
Home alone	3
Home with family member(s)	1
Retirement home/independent living	1
Assisting living/long-term care	4
Other	1
Relationship status	
Single	3
Married	2
Separated	1
Widowed	3
Other	1
Education	
Elementary school	3
High school or equivalent	5
College	1
Post-graduate degree	1
Vision devices	
Glasses	8
None	2
Hearing devices	
Hearing aid (both ears)	2
None	8
Major auditory/visual condition	
Total deafness	1
Deafness (one ear)	1
None	8
Head mobility	
Almost immobile	0
Limited	1
Normal	9
Body mobility	
Almost immobile	1
Limited	8
Normal	1
Mobility aids	
Cane	1
Walker	3
Wheelchair	3
Multiple mobility aids	3

^a^Mean (SD)

### Screening

All new admissions to general internal medicine (GIM) of patients over the age of 65 were screened by the research coordinator (RC). A number of potential participants were deemed “for review” after screening, and a further assessment of eligibility was performed by a study physician shortly after admission. The purpose of this review was to revisit the patient’s electronic medical record to look for additional documentation that might clarify if the patient was eligible for the study, in case a new diagnosis was made by a physician during the hospital stay, for example, a patient may be admitted with confusion and a history of memory impairment but has no evidence of a diagnosis of dementia documented in their EMR.

Potential participants were unknown to the RC prior to recruitment.

### Informed consent

This pilot study recruited patients deemed cognitively able to provide consent based on the assessment of the patient’s healthcare providers at the study site, and/or patients deemed unable to consent and have an SDM who can legally consent on the patient’s behalf. The consent process followed Ontario’s legislation [26], and the hospital provided a brochure [27] to help patients and families understand the different roles involved in decision making on one’s behalf. A previously documented capacity assessment determining that the potential participant is not competent to provide consent was respected. For potential participants for whom there was no record of capacity assessment, capacity for giving consent was obtained as per the study site protocol.

The SDMs of patients meeting the inclusion criteria were contacted over the phone to be introduced to the study. They were contacted by the RC, typically in the morning, following the screening process using an MGH office phone. SDM contact information was found in a distinct section of each patient’s EMR. The RC also reviewed the admission history and most recent physician-written progress notes to obtain the most up-to-date contact information for the SDM. In the event of discrepancies between the SDM contact information section of the EMR and the notes entered manually by physicians, the latter was considered more reliable and up-to-date.

The RC then obtained informed consent from SDMs in person after answering all questions presented over the phone and in person. In addition, the RC obtained assent from every participant at the beginning of each study session. Study sessions were scheduled at times that did not interfere with participants’ treatments or tests at the hospital.

### Intervention

**Fig. 1:**
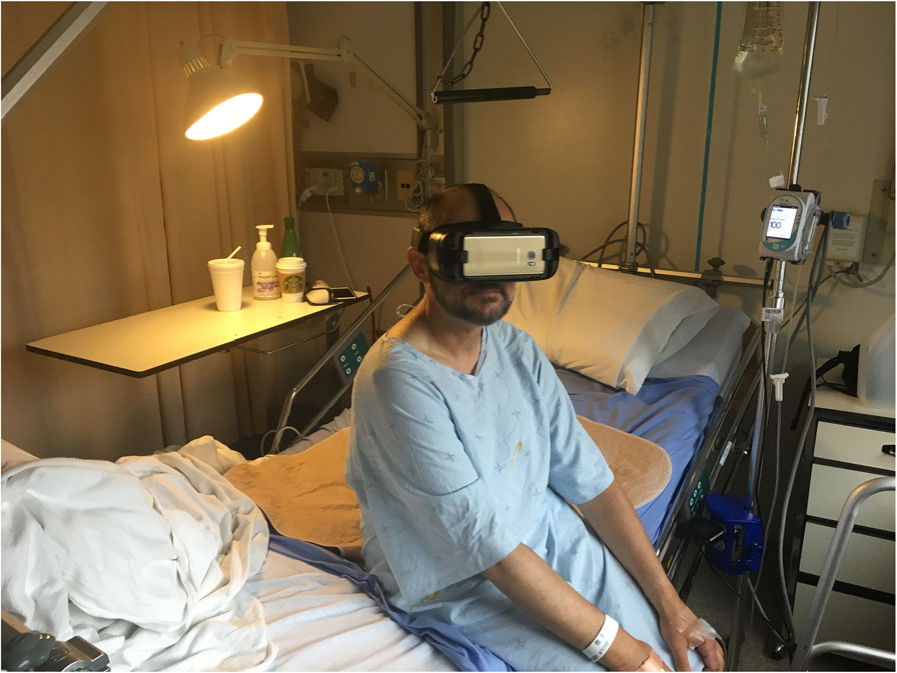
Example of participant trying the VR experience. A nurse, informal caregiver, or RC helped the participants sit up in their hospital room bed, and the RC helped them put on and remove the HMD and headphones. Written, informed consent was obtained from the individuals for the publication of this image

**Fig. 2:**
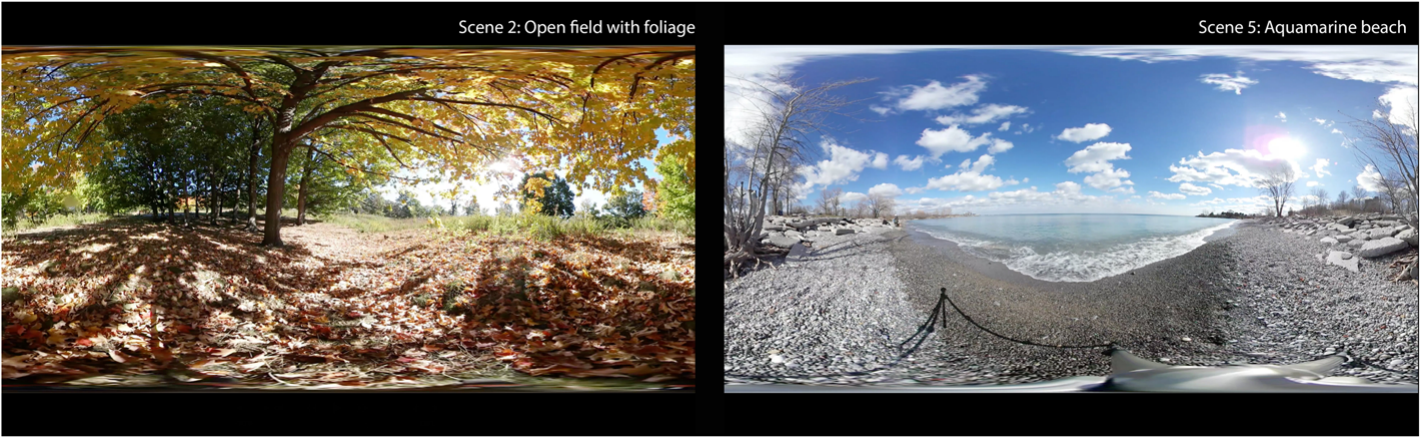
2D screen capture of two of the five VR scenes (Scenes 2: Open field with foliage and 5: Aquamarine beach). All participants viewed the same 360° VR experience, consisting of a sequence of five nature films

The intervention consisted of a VR session where participants viewed immersive VR experiences (VR films) for a maximum of 20 min; there was no minimum time requirement. Participants wore a Samsung Gear VR head-mounted display (HMD) and Sennheiser HD 221 headphones. The HMD was equipped with a personal removable hygienic foam insert purchased from VRology [28] for each participant to use throughout the study. A nurse, informal caregiver, or RC helped the participants sit up in their hospital room bed, and the RC helped them put on and remove the HMD and headphones (see Fig. 1). All participants viewed the same 360° VR experience, consisting of a sequence of five nature films (see Fig. 2) on a loop lasting a total of 6 min, as follows: 1 min of a rocky lakeshore, 1 min of a sunny forest, 1 min of a dense forest, 1 min of floating icebergs, and 2 min of a sunny beach.

### Data collection

#### Evaluation of study protocol feasibility (primary objective)

In an effort to capture a comprehensive and detailed summary of the research process including task sequence and timing requirements for each step, the research team recorded the time stamps at the beginning and end of each study task (e.g. time arriving on/leaving off the ward, time starting/stopping VR therapy). Qualitative observations made by the RC regarding hospital staff availability versus expectations / requirement, and other workflow difficulties encountered were also tracked by the RC (e.g. availability of the nurse/ward clerk, time to speak to the nurse/ward clerk, reason for not obtaining assent, delays related and unrelated to technology, and miscellaneous comments). Once the pilot was complete, the research team met to review the collected data and identified the variables affecting feasibility to an extent that could negatively impact the success of conducting the subsequent RCT. These variables were grouped into three categories: Processes, Materials, and Resources.

“Process” requirements refer to changes in research study tasks or the means and methods by which tasks are achieved, for example, the way in which screening patients for inclusion/exclusion criteria is conducted, how to contact effectively and efficiently the SDMs, and how to obtain informed consent in a more timely manner while ensuring an ethical process. Process requirements were further sub-divided into (1) screening and consenting tasks and (2) conducting the VR study sessions.

Elements categorized into “materials” requirements addressed changes to equipment or instruments, either related to the VR technology hardware (HMD) or software (films), or the study data collection (e.g. number of questions in the survey, amount of data points collected from the EMR).

Finally, “resources” requirements include the amount of time and materials required to complete tasks, personnel that need to be consulted (e.g. nurses, ward clerks), and elements of the hospital environment needed in order for the intervention to take place.

In this manuscript, we report on the findings and provide recommendations for changes to the research protocol to achieve a feasible RCT that evaluates VR therapy in the acute care hospital, focusing on the processes, materials, and resources.

#### Evaluation of VR intervention /proof-of-concept (exploratory objective)

The research team collected data from participants and their caregivers (if present) during study sessions using structured and semi-structured pre- and post-intervention questionnaires, observations following a guided script, and the 12-item Neuropsychiatric Inventory. Other participant data for the study were collected from the EMR, including physiological markers, delirium status, factors related to the acute care hospital care experience, length of stay, discharge disposition, in-hospital mortality, cognitive assessments, comorbidities, and general health history. The data collection tools and associated clinical outcomes are described in detail in a different article [23].

## Results

### Process

#### Screening

An average of 9.9 patients were admitted to the MGH GIM department every day of the study period; of these, a significant number (mean = 31, SD = 5.4) were on the first day of the work week. An average of 9.8 patients were screened every day of the work week, with the majority (46%) being screened on the first day of the work week. Average time to screen one patient was 4.1 min. Of the 516 patients admitted during the study period, 363 were over the age of 65. Of those, 271 screened not eligible for the study, 58 screened eligible, and 34 were deemed “for review”. The patients deemed “for review” were those admitted to the hospital without a previously established diagnosis of dementia and presenting symptoms that could be attributed to either dementia and/or delirium. After review, a total of 67 patients were eligible for the study. (see the Recruitment Flow Diagram (Additional file) for the overall results of the screening and enrolment process)

The study team observed during the pilot a screening limitation due to missed diagnoses of dementia in patient admission histories at the time of screening. For example, if a diagnosis of dementia was input into the EMR after the patient had already been screened by the RC, the patient represented a false negative (ineligible) screen and was excluded as study participant. With the available research staff (between one and three researchers) on site to complete all study activities, it was not feasible to re-screen all patients who initially screened not eligible; thus, a number of potential participants may have been lost to the study.

#### Capacity to provide consent

At the time of screening, all 67 prospective participants had either no previously documented capacity assessment or had a capacity assessment determining that they were not competent to provide consent. One prospective participant had a documented capacity assessment determining that they were competent to provide consent at their baseline, but not in their current condition. Upon speaking to an SDM, one prospective participant had the capacity to provide consent, which was confirmed by the potential participant’s nurse.

#### Informed consent

A total of 234 phone calls were initiated by the RC to the SDMs of the 67 eligible patients. A total of 101 of these calls (43%) resulted in an answering machine. When SDMs were not available to meet in person on the same day as the initial phone call, the research coordinator offered to email them the informed consent and assent forms, if they were willing to receive/review these documents.

Of the 67 eligible patients, 57 were not enrolled into the study, for the following reasons: the SDM declined participation (46%), the patient was discharged before the SDM could be contacted (25%), the patient was discharged before the SDM responded regarding participation (14%), SDM reported the patient did not have an official diagnosis of dementia (5.3%), the patient had been admitted previously and SDM had declined (1.8%), and the patient passed away (1.8%).

Of the 26 SDMs who declined participation for their patients, their reasons for decline were as follows: SDM believed the patient’s overall health at the time was too poor (27%), SDM was unable to physically come in to hospital (17%), SDM was not interested in the study/research (17%), SDM stated that the patient was not interested (12%), SDM thought the patient would not tolerate the headset (cataracts, irritability, poor reactions to touch) (7.7%), and significant SDM/patient language barrier (5.8%).

Of the ten patients for whom SDMs consented to participation and were enrolled, none withdrew early from the study. Four of the ten SDMs were sent electronic copies of consenting documents and were instructed to read the documents and prepare any questions/concerns they have about the study before meeting with the RC in person. The informed consent process with SDMs in person took an average of 14 min, and was considerably faster (by 19 min) when SDMs were emailed the consent/assent documents beforehand (average = 2.8 min) compared to when they were given the documents to read for first time at the hospital (average = 22 min).

#### VR sessions

Participants had their first study session an average of 5 days after being admitted to hospital. Four of the initial study sessions took place in the first 2 days after admission, three took place 3 to 6 days after admission, and three took place 7 or more days after admission. An average of 2.8 calls or call attempts to the participants’ SDMs were made in order to arrange the initial study session. For the large majority (9 participants), the informed consent was signed at the beginning of the initial study session. For one participant, the informed consent was signed 3 days before the initial study session occurred. This was because the planned initial study session was postponed 3 days due to the participant’s condition—the SDM stated that the participant “hasn’t opened [their] eyes for hours” and was not at their baseline because they had not slept the night before.

Upon entering the room for a VR session, participants were occasionally resting or asleep. If the participant did not rouse after the RC knocked on the door and called their name, the RC left the ward and returned at a later time.

The majority of participants (7) opted for additional sessions of VR therapy during their stay in hospital. Unfortunately, only three were actually able to participate in these additional sessions, while the remaining four could not continue to participate due to transfer to Complex Continuing Care (1), discharge from hospital (1), scheduling difficulties with SDM (1), and expiration (1). Therefore, the majority of participants (7) had only one (the initial) session of VR therapy. For additional sessions, the average length of time participants viewed VR films was 3.9 min.

The day of discharge is typically very busy with tasks that involve the patient and caregiver in patient-oriented summarization, such as information review regarding the hospitalization and discussion of important medication changes and chronic disease management points. In addition, for many patients with dementia, some form of home service organization and transportation arrangement is required. Conducting study sessions on the day of discharge proved to be difficult to schedule around these tasks and could have been disruptive to discharge planning.

### Materials

#### Data collection tools

All study data for the pilot were recorded on paper, then transferred to an Excel document that was updated iteratively.

##### Quantitative data

The average length of the initial study session (data collection and VR exposure) was 53.6 min. This was largely due to the administration of the NPI, which took an average of 19.5 min. The 12-item NPI was used as a baseline patient-specific metric of BPSDs and was administered to an informed caregiver, ideally an individual who lives and/or spends the most time with the patient. The informed caregiver was typically the same person as the SDM and was a family member of the participant in 9 out of 10 cases. The validated tool contains questions about changes in the patient’s behaviour that have appeared since the onset of dementia and have been present for the past 4 to 6 weeks. The average length of additional study sessions, in which the NPI was not collected, was 20 min.

Checking the participants’ current medical condition and daily schedule (heart rate, blood pressure, and blood glucose measurements) was not a timely endeavor, taking an average of 2.2 min and 1.4 min respectively. Certain clinical measures, like the daily Confusion Assessment Method (CAM) score that was used to determine presence of delirium, were not consistently reported. Similarly, Montreal Cognitive Assessment (MoCA) and Mini-Mental State Exam (MMSE) scores, which are used to assess cognitive impairment, were infrequently recorded. Moreover, various tools were used (e.g. one person had MMSE, MoCA, from various years, one person had one sub-scale from a mini-cog screen, one person had a depression measure)—and there was not enough overlap to meaningfully determine cognitive status even amongst the individuals who did have scores in their EMR. Therefore, we decided to categorize cognitive status based on the terminology used by physicians in their notes (mild, moderate, advanced).

Pilot data on instances of BPSD during the hospital stay were collected for each participant on hospital discharge, by examining the nursing notes and counting the total number of BPSD instances during patients’ entire hospital stay. First, the RC recorded and briefly described instances of BPSD using the terms recorded by nurses in the EMR. The team’s geriatrician then used this list to iteratively generate a set of categories to be used for recording instances of BPSD:

- Agitation,
- Refusing/declining medical care,
- Violent behaviour towards staff or other patients,
- Wandering,
- Vocalization,
- Insomnia,
- Mood symptoms,
- Disorganized thought or content,
- Perceptual disturbances,
- Additional falls precautions applied, and
- Sitter/PCS/PSW at the bedside.

Instances of BPSD were then collected again by the trained RC using these coded categories. When unfamiliar terms were found in the nurses’ notes, the RC consulted the geriatrician to correctly assign the instance to its corresponding category.

##### Qualitative data

We collected qualitative data relating to the participant’s VR experience using semi-structured interviews. Four participants could consistently respond reliably to the questions, one was able to respond in their first session and then had delirium halfway through their stay and was unable to respond reliably. The remaining five patients had difficulty answering questions about their mood before and after VR therapy. The RC often relied on caregiver input and participant body language to make educated estimations of participants’ moods. Some feelings/moods were impossible for observers to estimate (e.g. feeling adventurous) while others were usually possible (e.g. feeling tired, feeling energetic). Different sources of qualitative information contributing to the same outcome measure made statistical analysis challenging, as much of it was recorded in unstructured text as “Other Comments”. Additionally, the number of contributing sources varied between participants and across sessions within same participants.

#### Equipment/devices

##### Hardware/software performance

Of the 18 sessions conducted, technical difficulties were experienced in a minority (4) of sessions; two due to difficulty synchronizing the smartphone with the HMD, one due to the clips on the HMD falling off which made the phone fall out, and one due to difficulty reducing the volume of the audio (so in this case, headphones were not used, audio played directly from the phone).

Image quality was reported by participants as good at 11 of the sessions, for the remaining participants were not able to provide feedback. Sound quality was reported as good at 12 of the sessions; in five sessions, participants were not able to provide feedback; and in one session, the volume was too loud despite being played from the phone rather than through the headphones.

There were no difficulties in fitting the VR HMD and headphones in 16 sessions; in one session, the headphones slipped off, and in one session, the HMD was slipping down the participant’s face despite tightening the straps.

##### Safety, tolerability, and enjoyment

Clinical outcomes are reported in detail in a second paper, but overall, for the majority of sessions, participants were engaged while in VR, responding with some or substantial vocalizations. Researchers reported that at most sessions, participants appeared to enjoy the VR experiences and the majority resulted in relaxation. Only one of the ten participants experienced a negative side effect, which was minor, resulting in temporary feelings of dizziness and nausea. After the VR session, this participant was not averse to trying additional sessions, but their SDM decided against this.

Participants who were able to provide feedback (5) indicated that the VR content they would enjoy viewing in the future should include animals in nature, fields of flowers, the ocean, and Christmas-themed scenes.

### Resources

Resource requirements refer to any environmental needs and personnel that are not part of the study or the research team but are indispensable for ensuring the effective and efficient conduct of the study. This includes aspects of the patient’s hospital room (e.g. hospital bed, chairs), infection control materials available on the ward (e.g. disposable masks, hydrogen peroxide wipes), and hospital staff to be consulted by a member of the research team (e.g. nurse, ward clerk).

For the purpose of this study, the patient’s current condition was obtained from the primary nurse (the nurse assigned to the patient). This was done to ensure timely, accurate, and up-to-date information about the patient’s condition. If the participant’s primary nurse was not available, the next most responsible nurse was the “team lead”. Finally, if the team lead was not available, we resorted to asking for the nurse covering for the primary nurse. There was one occasion when finding a nurse responsible for the participant took several minutes due to multiple nurses being on break and the participant residing in a ward unfamiliar to the RC.

The nurse assigned to the patient was consulted prior to each session to ensure the patient is stable and they are otherwise able to participate in a VR session. The nurse was also asked for information pertinent to the researcher, which can include a safety check for aggressive or violent behaviours or planned care in the next half hour. The nurse was also consulted for information regarding changes from baseline that may indicate underlying delirium. Asking specific and directed questions were found to be the most effective and least time consuming for the nurse. Again, a proper introduction of the researcher’s position, role, and their request of the nurse upfront allowed the nurse to quickly understand the context. The questions that we found were most effective to elicit the information we were looking for included “Are the patient’s vitals stable?”, “Is there any reason why you think the patient could not participate in a VR session” (explaining what the session entails if needed), and “is there anything else we should know about this patient”.

Before each session, the ward clerk was also consulted to ensure the patient’s schedule was clear so that the VR session would not interfere with patient care.

Ward clerks were most receptive when researchers identified themselves as a research coordinator, with which study, and which local principal investigator. This was usually a quick process; factors slowing down this stage included the clerk being unfamiliar with the RC/study, or if the clerk was currently attending to the phone or another person. The latter cannot be avoided, but to improve the former, we found that a thorough introduction was helpful. Additionally, once known to the clerk, re-introduction before each session was not necessary.

## Discussion

### Process

#### Screening

As a significant number of new patients appear on the first day of the work week, we recommend that additional time is dedicated for screening that day.

A challenge that frequently arose during screening was determining the inclusion of patients admitted with “query dementia versus delirium”, who were potential participants without a formal diagnosis of dementia. The process used during the pilot for these patients (deemed “for review”) will be revised for the RCT, to include an additional step to make this determination, by requesting a geriatrics consult where appropriate. When the RC screens an individual without a previous diagnosis of dementia but who shows possible *signs* of dementia (ex. admitted with confusion, has a history of memory loss, has become less independent with ADLs/IADLs), the RC will contact the principal investigator (PI), an internist at MGH, who will review the patient’s history. If the PI determines that the patient may indeed have dementia, they will contact the patient’s most responsible physician (MRP) about this clinical question. If the MRP considers appropriate, they will then order a consult with a geriatrician to determine if a diagnosis of dementia can be made. If these patients receive a diagnosis of dementia, it expands the pool of potential study participants.

An additional screening limitation observed during the pilot was diagnoses of dementia omitted from patient admission histories or consult notes at the time of screening. This resulted in false negatives and excluded potential study participants. This issue was still unresolved at the time of designing the subsequent RCT.

Conducting the pilot highlighted new screening considerations due to potential interactions between the VR technology and common comorbidities. Thus, additional exclusion criteria will be introduced for the RCT in order to avoid unnecessary risk and focus on an appropriate patient population. For example, patients with a history of seizures or epilepsy will be excluded from the RCT based on the Oculus Go health and safety warnings which notes that some people (1/4000) experience seizures triggered by TV, video games, or VR [29]. Although the health and safety warnings note that these seizures are more common in children and young adults, clinical judgments of an internist and a geriatrician at the hospital (members of the research team) led the study team to place “patient history of seizures or epilepsy” on the list of exclusion criteria. For similar reasons, patients with a pacemaker will be excluded from the RCT. The Oculus Go health and safety warnings note that the headset and controller “may contain magnets or components that emit radio waves, which could affect the operation of nearby electronics, including cardiac pacemakers, hearing aids and defibrillators” [29]. They recommend that individuals should consult their doctor or the manufacturer of their pacemaker before using the headset or controller. The study team discussed the health and safety warnings and concluded that for the RCT, it is more appropriate to exclude patients with pacemakers since this was considered the safest approach (also it would be impractical to individually check with the MRP and/or the device manufacturer for each model of pacemaker).

Hearing aids are another medical device with potential for interference with the VR HMD according to the Oculus Go health and safety warnings [29]. However, due to low potential risk (i.e. transient static or whistling sounds) and previous research in this population finding no negative outcomes due to hearing aid interference, the research team chose to include patients with hearing aids.

Finally, patients with head trauma or stroke leading to the hospital admission will be excluded from the RCT due to possible light sensitivity, which can be triggered by the visuals in VR, and hemiparesis, which can affect their ability to experience VR and 360° movement.

#### Consenting

During the pilot, many SDMs were not available to come to the hospital in person; approximately 17% of all SDM declines to participate were due to the inability to physically sign the informed consent form, which was a requirement per the pilot study protocol. To ensure a timely consent process for the RCT, the team suggested adopting a verbal consent process as well as offering to email the informed consent/assent forms to the SDM. Emailing the documents will give SDMs time to review them at their leisure and, if they wanted to, time to discuss the study with other people/decision makers. Of note, the research team received ethics board approval to use a verbal consent script to obtain informed consent over the phone for the subsequent RCT.

The pilot study employed a shared model of consent, where the SDMs provided informed consent and participants assented to the intervention at the beginning of each session. An important finding from the pilot was that some participants were unable to verbally communicate their assent due to cognitive state and/or a language barrier. We recommended to revise the assent process for the RCT to record physical signs of assent/dissent when the participant cannot communicate verbally. The informed consent and assent forms for the RCT will be simplified and improved upon from a plain language perspective (Permission for reuse of these forms may be provided by contacting the authors). Important additions to the RCT informed consent form involve including descriptive pictures (VR HMD, disposable facial covers, sample nature scenes) and documenting the possibility of interference of the VR HMD with hearing aids.

A finding during the pilot was that the EMR field specifically designated for documenting the SDM and their contact information was not always accurate. For example, this field has been marked as “No SDM”, while the SDM’s info was recorded in the “alternate contact” field. In another couple of instances, family members have been disputing SDM/POA status. Ultimately, we found that the physician consult notes were more accurate. We recommend for the RCT that the RC consult the most recent physician notes from the current admission to ensure they are contacting the appropriate decision maker. Also, if the SDM is not available upon the first contact attempt, the RC should identify and attempt to contact the Alternate Contact (if applicable).

Although SDM language barrier was not a common reason for non-participation, there were several cases where the SDMs with language barriers gained basic understanding of the study with the help of other family members who could translate for them. It is important to check and confirm with the hospital’s ethics board whether another family member can help the SDM translate informed consent materials from English into their language.

More than a quarter of all “declines” were due to the SDM believing the patient’s current state of health was too poor. For the RCT, to ease these concerns, the research team will report the results of this pilot study, where the headset was well tolerated by all participants including the acutely ill patients. The RC will be advised to also direct SDMs to the study’s website [30] that provides additional information and demonstrations of the VR device.

#### VR sessions

During the pilot study, if upon entering the room for a VR session, participants were resting or asleep and did not rouse when the RC called their name, the RC left the ward and returned at a later time. After reviewing this approach for the RCT, the research team decided that, in order to promote activity during the day in an effort to reduce wakefulness and wandering overnight, participants should be woken up if they are asleep when the RC visits them for the study sessions. Before VR therapy, participants will be asked questions like “How are you feeling today?” and “How did you sleep?” to gauge their mood and stimulate a conversation with the RC.

During the pilot, it was very difficult to schedule study sessions on the day of discharge and was potentially disruptive to discharge planning. For the RCT, the research team decided to not knowingly conduct study sessions on the day of discharge.

Finally, of the seven participants who opted for additional sessions of VR, only three were actually able to participate in additional sessions. For the RCT, some of the obstacles will be removed by adopting verbal informed consent from the SDMs, which will likely result in a faster enrolment process and therefore starting the study sessions earlier into the hospital admission.

### Materials

#### Data collection tools/instruments

For the pilot, we collected and analyzed data using MS Excel. While this approach worked for the small size pilot sample, a critical requirement made for the RCT was to collect/record all study data using a secure web application specifically designed to manage research databases that complies with local personal health information storing and sharing standards (e.g. HIPPA). All efforts should be made to eliminate redundant data collection and reduce the likelihood of human error due to repeated data entry.

As CAM scores were performed on an inconsistent basis and did not always accurately reflect a patient’s delirium status, the study team decided to abandon the CAM in favour of physician notes from the EMR and nurse impressions of delirium progression before each session. Similarly, because the severity of dementia (e.g. mild, moderate, advanced) was infrequently found in the EMR, for the RCT this will be determined from a combination of any available cognitive assessment scores (e.g. MoCA and MMSE) and dementia severity if recorded in the EMR.

Recording all heart rate, blood pressure, and blood glucose measurements taken during the hospital stay for the pilot study participants proved to be superfluous. Changes recommended for the RCT were as follows: blood glucose will no longer be recorded as it is very infrequently measured in patients without diabetes and would be difficult to claim statistical significance without controlling for mealtimes. Additionally, heart rate and blood pressure measurements will only be recorded twice daily, once around 09:00 and once around 16:00, to provide a manageable data set that can be used to establish vital sign trends for both arms of the RCT.

Significant modifications were recommended for the instruments used to detect changes in BPSD during the hospital stay. Firstly, new categories were created by the team’s geriatrician during the pilot, to help group BPSD instances collected from the nurses’ notes in the EMR. These categories will be used to determine the types and frequencies of BPSDs in the subsequent RCT. For the RCT, the study team also recommended to replace the NPI with the E-BEHAVE-AD [31]. Although caregivers from home may be able to give informed feedback at the beginning of the hospital stay, they likely cannot spend enough time at the hospital to reflect on changes during the patient’s stay. Also, with the addition of a remote (verbal) informed consent process, the RCT is likely to include caregivers with very limited time available to visit the patient. A variation of the NPI, the Neuropsychiatric Inventory - Nursing Home (NPI-NH), is administered to professional caregivers (e.g. nurses) but was designed for patients in extended care facilities rather than acute care. The E-BEHAVE-AD is an observational evaluation of BPSD following a brief 20-min conversation with the patient. It can be conducted by a member of the research team, has high inter-rater reliability, and requires minimal training. The use of chemical and physical restraints, collected from the EMR, will also be used to detect changes in BPSD during the hospital stay.

Finally, we found that 60% of the pilot participants had difficulty answering questions about their mood before and after VR therapy, and the RC often relied on caregiver input and participant body language to make educated estimations of participants’ moods that they recorded as being communicated by participants themselves. To overcome these challenges in the RCT, the study team will replace this combination of participant-caregiver-research coordinator-reported outcomes about feelings and mood with distinct questions targeted at each source (i.e. participant, or RC). Participants will be asked fewer questions in total with a greater focus on simple, open-ended queries. The Smiley-Face Assessment Scale will be provided if the participant has difficulty verbally expressing themselves. This conversation with the participant will provide the RC with time to observe the participant and score the E-BEHAVE-AD and In-Hospital Quality of Life Observational Scale (adapted from the Quality of Life in Late-Stage Dementia (QUALID)). Relevant, unprompted caregiver comments will be recorded separately.

Lastly, as per the suggestions of the participants, we will expand our VR film offering for the RCT to include a greater diversity of experiences that they can request at each session. For example, we will add live music scenes featuring classical music, scenes featuring people walking around, and scenes featuring animals.

#### Equipment/devices

Delays related to the VR devices were largely due to difficulty synching the phone and the HMD. To avoid this issue and for other ease of use reasons, for the RCT we plan to use the Oculus Go HMD. Unlike the Samsung VR HMD used for the pilot, the Oculus Go is a standalone device that does not need an attached cell phone to act as a screen. The Samsung Gear VR HMD was well tolerated by the participants and was reported to be comfortable by seven out of ten participants (2 participants were unable to answer). The Oculus Go HMD is predicted to be equally or more comfortable and well-tolerated as it is also more glasses-friendly and weighs even less than the Samsung Gear VR HMD (468 g and > 500 g respectively).

Few challenges arose related to the HMD comfort and fit. There were two sessions where the adjustable head straps were ill-fitting and resulted in the HMD slipping down the participant’s face. Compared to the Samsung Gear VR, the head straps on the Oculus Go are larger with greater support for the back of the head. We predict that this will help prevent the issue of the HMD slipping down the face.

From an infection control perspective, the Oculus Go HMD differs from the Samsung Gear VR HMD in that the default facial interface is porous fabric as opposed to non-porous plastic. The study team purchased an “Oculus Go Starter Pack” from the company “VR Cover” which contains a wipeable and machine-washable custom facial interface, a wipeable polyurethane leather facial cushion, and disposable stick-on hygiene covers. The facial interface and facial cushion will be wiped with hydrogen peroxide wipes available on the ward. The HMD head straps will be replaced if/when they wear down. The study team consulted the MGH Infection Prevention and Control (IPAC) team and received approval to use these products when sharing the HMD across all patients, even those with infection control precautions in place.

Only one of the ten participants in the pilot study experienced dizziness and nausea due to the VR films. Such symptoms can likely be avoided by moving the head slower, and fixating the eyes on one point of the VR environment while turning the head. In the RCT, these recommendations will be communicated to participants before or during the VR session as necessary.

### Resources

An observation related to resources that needs emphasis for the RCT is the importance for research staff to introduce themselves to ward staff, for several reasons, including security, and to create a good rapport. Nurses are urged to ask people they do not recognize for identification, so research staff can be proactive by offering this information. To avoid delays related to the ward staff workflow, we found that it was helpful to become familiar with the unit including the clerks, nurses, and their schedules. When known, it is preferable to avoid visiting wards during nursing break times.

### Outstanding issues (identified and still needing resolutions)

While the authors provide suggestions that address the majority of issues that arose during the pilot trial, a number of challenges remain. A screening limitation that was observed during the pilot for which no change has been suggested yet, was the missed diagnoses of dementia in patient admission histories. For example, if a diagnosis of dementia is input into the EMR after the patient had already been screened by the RC, the patient would be a false negative (i.e. ineligible for participation in the study). Without re-screening, all patients initially screened negative—which was found not feasible—a number of potential participants may be lost to the study.

During the pilot, close to half of all calls made by the RC to SDMs resulted in an answering machine. Unfortunately, this is an unavoidable barrier to recruitment, as it is not best practice to leave voicemail messages for research purposes.

## Limitations

As this was a pilot study with the goal of refining the research protocol for the subsequent RCT, it only included ten patients, and thus findings and suggestions must be interpreted with caution. Furthermore, the randomization protocol was not tested and may introduce new challenges that have not been uncovered in the pilot.

The hospital processes, tools, and resources used in this study are those of MGH, a community teaching hospital located in Toronto, Canada. Although many of the general principles are generalizable to other acute care hospitals, some practices/processes may be very specific to this hospital and not applicable to other settings. Introducing non-pharmacological therapeutic interventions should be tried and evaluated in different other acute hospitals to ensure generalizability of the outcomes.

Another difficulty encountered in the pilot was related to participants not being able (for various reasons) to have additional VR sessions beyond the initial one, even though they have opted for more VR sessions. Therefore, although the intent of the study was to measure outcomes from a number of VR sessions during their hospital stay, the majority of participants had only one session of VR therapy.

## Conclusions

VR technology has shown promise in healthcare in phobia management, pain reduction treatment, and physical/neuro-rehabilitation; while there are reports on the benefits of using VR with individuals with dementia, it has not been widely used or rigorously evaluated as a therapeutic intervention for managing BPSD and improving Quality of Life. This pilot study showed that overall, patients with dementia appear to tolerate immersive VR, and that it is feasible to evaluate this non-pharmacological intervention in acute care hospitals. The findings from our pilot study identified issues and brought specific suggestions for the design and conduct of the RCT, which will be the first to rigorously evaluate the impact of immersive VR therapy using HMD, with patients at all stages of dementia, in an acute care hospital setting.

Any deployable, scalable, non-pharmacologic solution to BPSD would go a long way helping dementia patients and their caregivers. While VRx may be one answer, this pilot has also provided methodological foundations that could be used for introducing and evaluating other non-pharmacological therapies into acute care hospital settings, as well as across other conditions, such as delirium, mild cognitive impairment, and stroke.

## Supplementary information

**Additional file 1.**

## Data Availability

“Not applicable”

## Abbreviations

ADL: Activities of daily living (Katz Index of Independence)
IADL: Instrumental Activities of Daily Living (Lawton Scale)
BPSD: Behavioural and psychological symptoms of dementia
CAM: Confusion Assessment Method
EMR: Emergency room
GIM: General internal medicine
HMD: Head-mounted display
IPAC: Infection Prevention and Control
MGH: Michael Garron Hospital
MMSE: Mini-Mental State Exam
MoCA: Montreal Cognitive Assessment
MRP: Most responsible physician
NPI: Neuropsychiatric Inventory
NPI-NH: Neuropsychiatric Inventory - Nursing Home
PI: Principal investigator
POA: Power of attorney
QoL: Quality of Life
QUALID: Quality of Life in Late-Stage Dementia
RC: Research coordinator
RCT: Randomized controlled trial
SDM: Substitute decision maker
VR: Virtual reality
