# Supplementary material for: Introducing virtual reality therapy for inpatients with dementia admitted to an acute care hospital: learnings from a pilot to pave the way to a randomized controlled trial
Source: Pilot Feasibility Stud. 2020 Oct 31;6:166. doi: 10.1186/s40814-020-00708-9 (PMC7602317; doi:10.1186/s40814-020-00708-9)
Supplement: Supplementary file 1 — Additional file 1. [file 40814_2020_708_MOESM1_ESM.doc]

**Additional Files**

Recruitment Flow-Diagram

**n= 10** Eligible participants consented into the study

**n= 516** Patients admitted during study period and assessed for eligibility

Excluded from recruitment

- **n= 153** aged under 65 years old
- **n= 271** did not meet other inclusion criteria

**n= 67** Met all inclusion criteria and were eligible for participation in the study and asked for consent

**n= 58** Met all inclusion criteria

**n= 34** Needed additional diagnosis review because admitted with ‘query dementia versus delirium’

**n= 25** Excluded due to delirium diagnosis

**n= 57** Patients (or SDMs) did not provide consent to participate in study
